# Habitat overlap between Asiatic black bear *Ursus thibetanus* and red panda *Ailurus fulgens* in Himalaya

**DOI:** 10.1371/journal.pone.0203697

**Published:** 2018-09-06

**Authors:** Manjit Bista, Saroj Panthi, Sarah R. Weiskopf

**Affiliations:** 1 Ministry of Forests and Environment, Department of National Parks and Wildlife Conservation, Babarmahal, Kathmandu, Nepal; 2 Ministry of Forests and Environment, Department of Forests, Babarmahal, Kathmandu, Nepal; 3 U.S. Geological Survey, National Climate Adaptation Science Center, Reston, VA, United States of America; Sichuan University, CHINA

## Abstract

Studying habitat overlap between sympatric species is one of the best ways to identify interspecies relationships and to direct conservation efforts so that multiple species can benefit. However, studies exploring interspecies relationships are very limited in Nepal, making it difficult for the government of Nepal and conservation partners to manage wildlife in their habitats, especially in Himalayan protected areas. In this study, we identified habitat overlap between Asiatic black bear (*Ursus thibetanus*) and red panda (*Ailurus fulgens*) as well as important habitat types for both species in the Makalu Barun National Park, Nepal using Maximum Entropy (MaxEnt) modeling. GPS points of species occurrence were collected from the field, and environmental variables were extracted from freely available sources. We found that the study area contained 647 km^2^ of Asiatic black bear habitat and 443 km^2^ of the red panda habitat. 368 km^2^ supported both species, which constituted 57% of the Asiatic black bear habitat and 83% of the red panda habitat. We found that conifer forest was the most important habitat type for both species. Because the largest portions of both species’ habitat were located inside the buffer zone, a peripheral zone of national park, conservation efforts for these sympatric species should be focused inside the buffer zone to be most effective.

## Introduction

Identifying species’ habitat preferences is essential for effective conservation. Managers need to know the type and condition of the habitat where species thrive to ensure that it is well managed. Habitat conditions in Nepal are declining due to people’s dependence on forests [1]. In the Himalayan region of the country, most carnivores avoid bare lands (rocky and non-vegetated areas) and areas with high anthropogenic pressure [2]. Lack of information regarding species habitat choices is hindering conservation efforts in the region. While several individual species-specific studies have been conducted in Nepal [3], very few studies have identified multiple species relationships.

One species of conservation concern in Nepal is the Asiatic black bear (*Ursus thibetanus*), which is native to Nepal and 19 other Asian countries [4]. It is listed as vulnerable by the International Union for Conservation of Nature red list [4] and is listed in Appendix I of Convention on International Trade in Endangered Species of Wild Fauna and Flora [5]. The species prefers mixed temperate oak (*Quercus semecarpifolia*) forests in Nepal [6]. It has been recorded between 1600 m to 3200 m in central Nepal [7], although its preferred elevation, at least in some areas, is between 2500 m and 3000 m [8], and its altitudinal limit is 4300 m [4]. The Asiatic black bear is facing anthropogenic pressure across its range, including habitat loss and fragmentation, poaching, and capture of bear cubs for sale [9,10]. In addition, human-bear conflict exacerbates existing threats. Asiatic black bears can cause major damage through livestock and crop (mainly maize) depredation, and may also attack humans [11–14]. In Nepal, bears were responsible for 12% of all wildlife encounters that resulted in death or injury between 2010 and 2014 [15].

Another species of conservation concern is the red panda (*Ailurus fulgens*), an endangered species native to five countries of Asia: Nepal, China, India, Bhutan and Myanmar [16]. It is one of the 26 protected mammals under Nepal’s National Parks and Wildlife Conservation Act [17], is listed in Appendix I of Convention on International Trade in Endangered Species of Wild Fauna and Flora [5], and is listed as endangered by the International Union for Conservation of Nature red list [16]. It prefers temperate evergreen forests where bamboo is the major ground cover, as the leaves and young shoots of bamboo are an important food source for the species [18–28]. Although it is protected by national and international laws, the population is declining due to habitat fragmentation and anthropogenic pressure [16]. The anthropogenic impact on its habitat has been identified as the major threat to the conservation of the species [29–32]. For example, hunting, habitat fragmentation, and conversion of natural forests into plantations are the major threats in China and India [33,34]. Additionally, cattle, herders and their guard dogs use the same habitat as the red panda in Nepal, which disturbs their natural habitat and has been directly attributed to red panda deaths [35].

Although both species are conserved by national and international laws and conventions, both face serious anthropogenic pressures [9,10,29]. Both Asiatic black bear red panda have similar altitudinal ranges [4,7,8,18,22]. Asiatic black bear in Manaslu Conservation Area of Nepal prefer mixed oak forest with associated broadleaved species (i.e., *Quercus lanata*, *Q*. *leucotrichophora*, *Q*. *lamellosa*, *Q*. *semicarpifolia*, *Juglans regia*) [6]. Similarly, the red panda is a habitat specialist, and prefers temperate forests with bamboo ground cover [18–20,22–24]. Bamboo is major feeding species of both Asiatic black bear and red panda [22,36].

Studies elucidating habitat overlap among multiple species can help managers develop protection strategies for multiple species of conservation interest at once [37–39]. While studies related to the distribution, diet, habitat, and threats of the Asiatic black bear and red panda have been conducted individually, no studies exploring their relationship, including habitat overlap, have been conducted [6–13, 19,20,22,23,25,27,29–31,35,40–42]. Due to the similarity in habitat, distribution, diet, and threats, it is useful to identify the spatial habitat overlap between these species and to identify major habitats to focus conservation efforts. This would allow the government of Nepal and conservation partners to protect parts of the study area, including areas of the park buffer zone that are not protected as effectively as the core zone of the national park, where both species can survive, allowing for the simultaneous conservation of the two threatened fauna. Therefore, the major objectives of our study were to (a) understand the quantity of overlapping habitat of these two species in the study area; and (b) determine which habitat types are used the most by these species. We predicted that the Asiatic black bear and red panda have overlapping habitat because they occupy similar habitat types and altitudinal ranges [4,6,9,18–24]. In this study, we used a Maximum Entropy (MaxEnt) model to determine suitable habitat for both species. MaxEnt is a widely used model to identify suitable habitat using species occurrence data and environmental variables, and is an established tool to explore spatial habitat overlap between multiple species [37, 43–51].

## Methods and materials

### Study area

**Fig 1:**
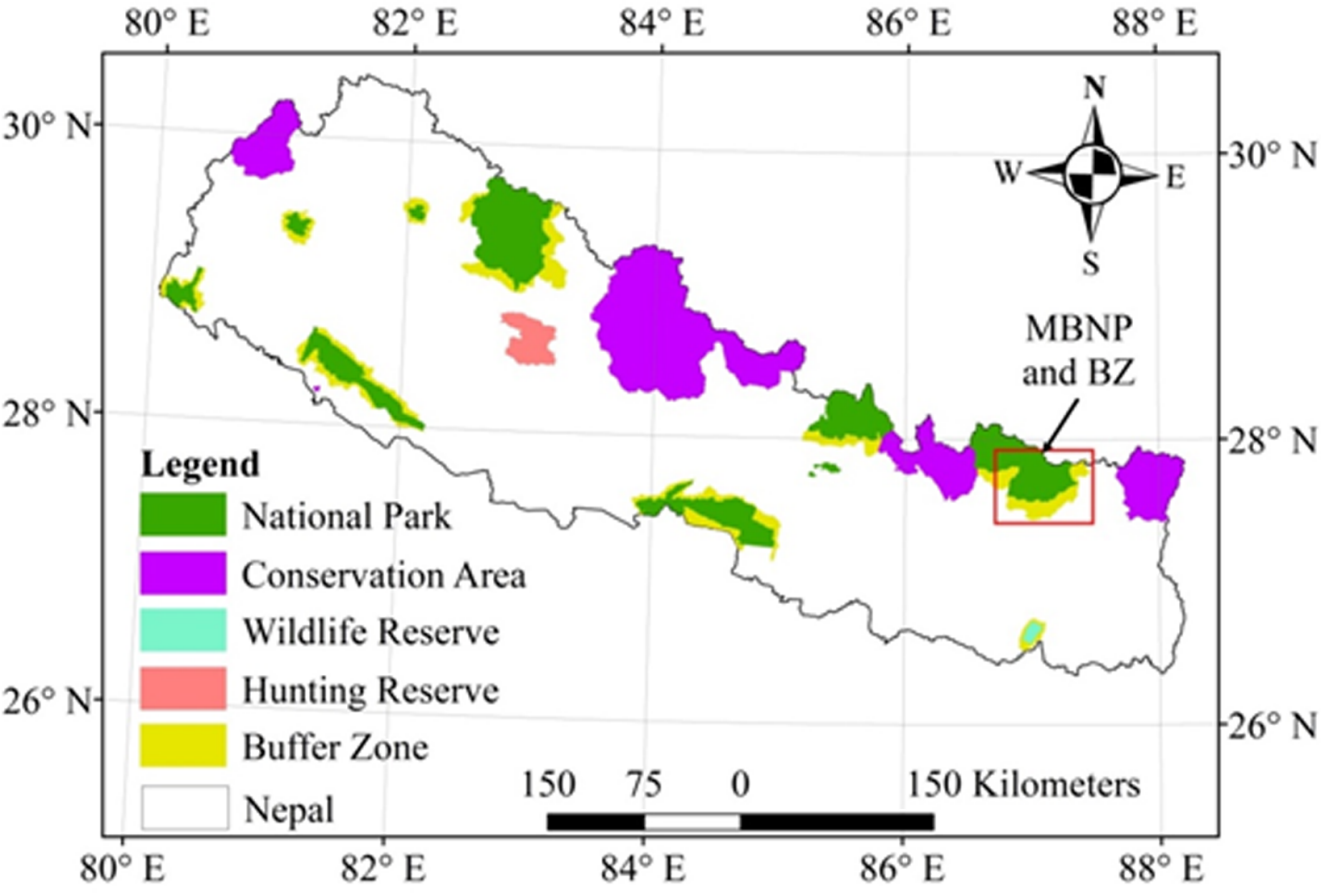
Protected areas of Nepal and the location of Makalu-Barun National Park (MBNP) and its Buffer Zone (BZ). (source of shape file: UNEP-WCMC & IUCN, 2017).

This work was conducted in Makalu-Barun National Park and its surrounding buffer zone in the eastern Himalaya region of Nepal with appropriate research permission (530-2071/2072; 542-2072/2073) from the Makalu-Barun National Park office, a field office of Department of National Parks and Wildlife Conservation for research (**Fig 1**). This national park was established in 1991, and covers 1500 km^2^. To manage the needs of local people and to minimize human-wildlife conflict, an 830 km^2^ buffer zone of the national park was created in 1999 in the areas where the forests were most useful to people [52]. The park supports diverse vegetation, ranging from tropical forest to alpine grassland, and is recognized for its tremendous biodiversity. The park harbors 25 species of rhododendron, 47 types of orchids, and 56 rare plants [53]. *Shorea robusta*, *Castanopsis* spp., *Quercus lamellosa*, *Q*. *semicarpifolia*, *Alnus nepalensis*, *Acer campbelli*, *Betula utilis*, *Rhododendron* spp., *Tsuga dumosa* and *Abies spectablis* are the major tree species of the park [54]. The major fauna of the park are snow leopard (*Uncia uncia*), red panda, musk deer (*Moschus chrysogaster*), Asiatic black bear and wild boar (*Sus scrofa*) [53].

### Data collection

We conducted informal interviews with local people and staff of the national park to identify potential habitats of the Asiatic black bear and red panda within the park and its buffer zone. Then, between May 2015 and June 2016, the first author and two other staff of Makalu-Barun National Park visited the potential habitats of these two species identified during the interviews. We visited each location twice to search for species occurrence, once in May or June and once in October or November to try to capture any seasonal change in habitat use. Where we saw the species or its scat, we recorded it as an occurrence. The scats of Asiatic black bear were identified by the experienced staff of Makalu-Barun National Park. No other bear species live in the altitudinal range where data were collected, and thus it was not difficult to identify the scat. The scats of red panda were identified by comparing them to photos of this species’ scats from Panthi [22] and with the help of experienced staff of Makalu-Barun National Park. We recorded the location of red panda (n = 66), and Asiatic black bear (n = 64) presence with a GPS receiver.

### Environmental variables

We used topographic, vegetation-related and anthropogenic variables for modeling suitable habitat (**Table 1**). We selected variables suspected to influence species presence based on the existing literature and expert opinion from the field. We used ArcGIS and ENVI software to process the variables [55–58]. We created a raster file with 30 m resolution of all environmental variables to fit the MaxEnt, a species distribution model. Because high resolution climatic variables are not available in this area, we used elevation as a proxy of temperature.

**Table 1:** Environmental variables used for modeling.

Source	Category	Variable	Abbreviation	Unit
U.S. Geological Survey	Topographic	Elevation	elevation	m
		Aspect	aspect	Degree
		Slope	slope	Degree
GEOFABRIK		Distance to water	dist_water	m
MODIS	Vegetation-related	Annual minimum EVI*	evimin	Dimensionless
		Annual mean EVI*	evimean	Dimensionless
		Annual maximum EVI*	evimax	Dimensionless
		Standard deviation EVI*	evisd	Dimensionless
Global Forest Change		Forest Cover	forest	Dimensionless
GEOFABRIK	Anthropogenic	Distance to settlement	dist_settle	m
		Distance to path	dist_path	m
International Centre for Integrated Mountain Development		Land use/land cover	landcover	m

* EVI = Enhanced Vegetation Index

### Topographic variables

We selected elevation, aspect, and slope as variables for our model, as these are the most important topographical factors impacting habitat selection by terrestrial animals [20,58]. We downloaded a 30 m resolution Digital Elevation Model (DEM) from the U.S. Geological Survey (USGS) website (https://earthexplorer.usgs.gov/), and calculated slope and aspect from the DEM. Both Asiatic black bear and red panda are terrestrial mammals but they need water for survival. We downloaded the shapefile of waterways from the Geofabrik (https://www.geofabrik.de/data/shapefiles.html) website and converted it to a distance raster file with ArcGIS [55].

### Vegetation-related variables

Vegetation is another major component of an animal’s habitat. Although Asiatic black bears and red pandas are carnivorous, they eat plants and live in forests, so vegetation-related variables are important to include when modeling their habitat [22,59]. Wang et al. [60] found a positive correlation between understory bamboo and the satellite-derived Normalized Difference Vegetation Index (NDVI), and Panthi [58] used NDVI to model the habitat of red pandas in Nepal. Similarly, Sun [61] used the Enhanced Vegetation Index (EVI) to model the habitat of the giant panda (*Ailuropoda melanoleuca*), a similar species to the red panda, in China. Therefore, we used EVI as a surrogate for understory bamboo. We chose EVI rather than NDVI for our model because EVI has improved sensitivity in high biomass regions. We downloaded EVI time series data from 2015, 2016, and 2017 from the Moderate Resolution Imaging Spectroradiometer (MODIS) sensor from the USGS website (https://earthexplorer.usgs.gov/). We then used ENVI software to smooth the data by using the upper envelope to reduce the cloud effect, and subsequently to obtain mean, maximum, minimum and standard deviations of EVI. We also used forest cover as a variable for the model. We downloaded forest cover data prepared by Hansen et al. [62] from the Global Forest Change website (GFC; http://earthenginepartners.appspot.com/science-2013-global-forest).

### Anthropogenic variables

Local people reside inside the buffer zone and frequently carry out activities, such as grazing livestock, hill slope burning, and collecting forest products, in the core area of the national park [63,64]. Significant human influence was observed in the park while we collected data. Therefore, anthropogenic variables were added to the model. We downloaded the shapefile of paths inside the study area from the Geofabrik (https://www.geofabrik.de/data/shapefiles.html) website. Settlement locations were available from Department of Survey, Nepal. We created distance raster files of paths and settlements using ArcGIS [55]. We also included data related to land cover and land use, which we downloaded from the website of International Centre for Integrated Mountain Development website (ICIMOD; http://www.icimod.org) [65].

### Modeling

We used MaxEnt version 3.4.1 (http://biodiversityinformatics.amnh.org/open_source/maxent/) to model suitable habitat of both Asiatic black bears and red pandas in the study area. The species occurrence points and environmental variables described above were used as model inputs. Multicollinearity between environmental variables described in **Table 1**were weak (|r|<0.70) in this case, so we used all variables in the model. At least 500 m distances between species presence points were maintained to reduce spatial autocorrelation. We selected ten replicates, and 1000 maximum iterations during the modeling [66].

Assessment of accuracy is essential to validate models and to understand model performance. We allocated 70% of the species occurrence points for the training dataset, and used 30% as a testing /validation dataset for both models. The models were evaluated by two methods, one threshold independent, and the other threshold dependent. In the threshold independent method, accuracy values were obtained directly from model, but in the threshold dependent method, we provided the threshold to maximize the sum of sensitivity and specificity. In the threshold independent method, we calculated the area under the receiver-operator curve (AUC) of the models [43,67]. A higher AUC signifies higher model performance. An AUC <0.7 denotes poor model performance, 0.7–0.9 denotes moderately useful model performance, and >0.9 denotes excellent model performance [68]. Although AUC is a widely used model evaluation parameter, it has been criticized by some researchers because it is influenced by the geographical extent over which models are carried out [69]. Therefore, we also used True Skill Statistic (TSS), a threshold dependent accuracy assessment, for model evaluation [70]. TSS = Sensitivity + Specificity − 1, and ranges from −1 to 1, where 1 indicates a perfect fit, and values less than 0 indicate a performance no better than random [71]. We calculated TSS for all model outputs, and final TSS was averaged from all ten replications [58]. The threshold to maximize the TSS is recommended for species distribution models which have presence-only data [72] so we used this threshold to convert the continuous probability map to a suitable/unsuitable binary map.

After running the models using the all variables described in **Table 1**, we converted the continuous habitat suitability map to a suitable/unsuitable binary map. We overlaid the maps of suitable habitats of both species in ArcGIS to delineate the overlapping habitat of the two species. We also overlaid the suitable habitat maps of both species and a map of land cover types in ArcGIS and determined the amount of suitable habitat covered by different land cover types to determine the most used habitat for each species.

## Results

### Habitat of Asiatic black bear and red panda

**Fig 2:**
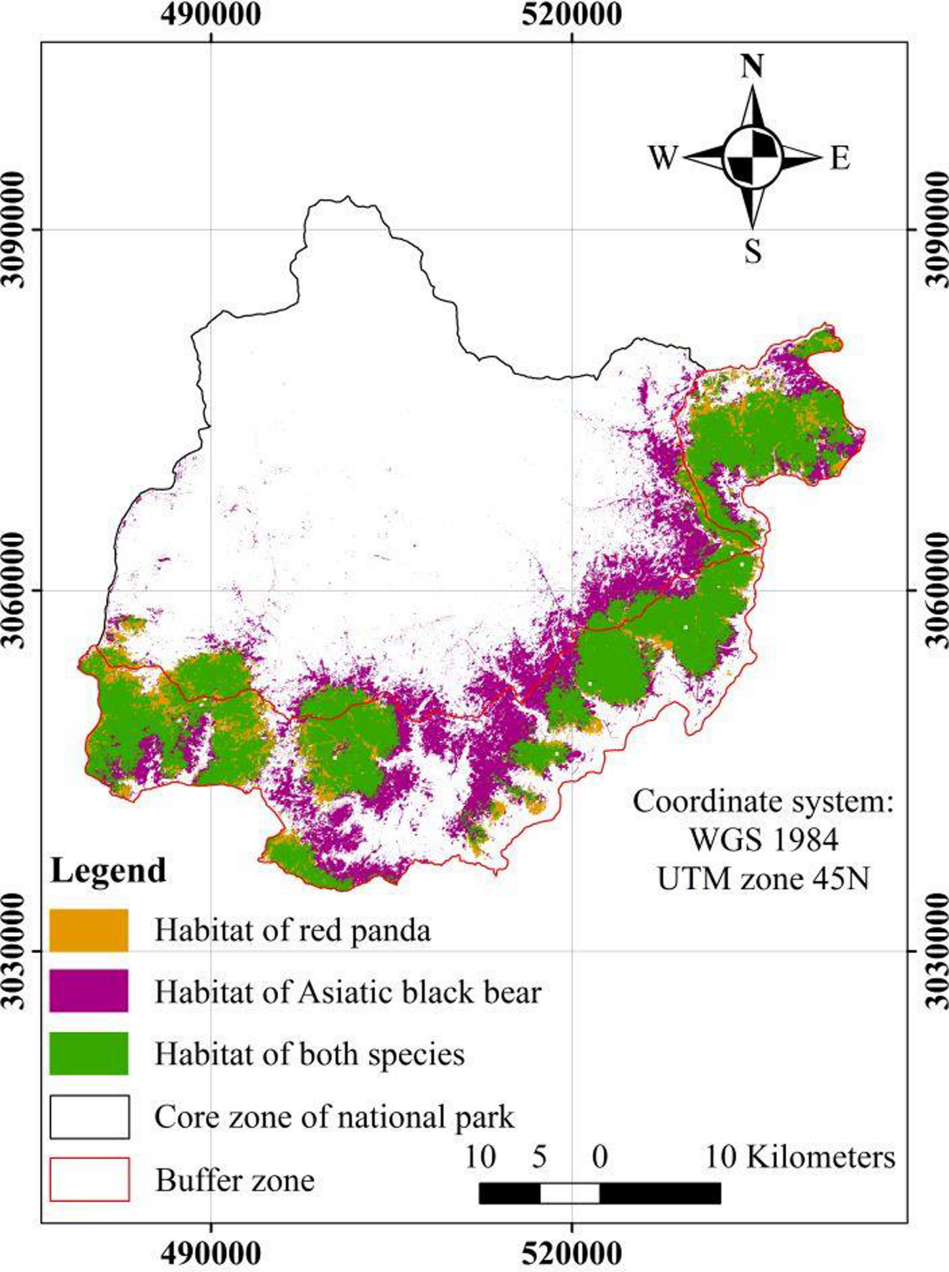
Habitat overlap between red panda and Asiatic black bear.

We found 647 km^2^ of suitable habitat for Asiatic black bear and 443 km^2^ of suitable habitat for red panda throughout the study area (**Fig 2**). The buffer zone contained 484 km^2^ and 380 km^2^ of the suitable habitat of the Asiatic black bear and red panda respectively. Remaining potential habitat was covered by the core zone of the national park. We identified 368 km^2^ of overlapping habitat between the species, which constituted 57% of the habitat of Asiatic black bear and 83% of the habitat of red panda. Most of the overlapping habitat was located in the southern and eastern parts of the study area, with a 318 km^2^ area located inside the buffer zone and a 50 km^2^ area located inside the core zone of the national park.

We found good AUC for the model of Asiatic black bear (0.800+/-0.025) and excellent AUC for the red panda (0.913+/- 0.019) habitat suitability model. The thresholds (0.237 for Asiatic black bear and 0.165 for red panda model) to maximize the sum of sensitivity and specificity were used to calculate the TSS and to convert the continuous probabilistic map to a binary suitable/unsuitable map. TSS of models of Asiatic black bear and red panda were 0.511+/-0.057 and 0.695+/-0.241, respectively.

### Habitat types in Asiatic black bear and red panda habitat

The majority of the study area is covered by forests, followed by snow/glacier and shrub land, but the majority of both Asiatic black bear and red panda habitat is covered by forests, followed by shrub land, grassland, and agricultural land (**Table 2**). Conifer forest covered more of the suitable habitat for both species than broadleaved forest. Only very small proportions of the habitat of both species fell on bare land, snow/glacier, and built up area. Rivers and lakes were not present in the suitable habitat of either species.

**Table 2:** Habitat types covering suitable habitat for Asiatic black bear and red panda.

		Area (km^2)^		
Habitat type/Land cover	Total Area	Asiatic black bear habitat	Red panda habitat	Habitat of both species
Conifer forest	632	417	276	247
Broad-leaved forest	208	72	52	40
Shrubland	300	88	60	44
Grassland	294	25	20	10
Agricultural land	70	36	28	24
Bare land	294	6	3	1
Built-up area	2	1	1	1
River	1	0	0	0
Lake	2	0	0	0
Snow/Glacier	527	3	2	1
Total	2330	647	443	368

## Discussion

Both Asiatic black bears and red pandas can share habitat with other species with similar characteristics. Asiatic black bears share habitat with sun bears (*Helarctos malayanus*) in Thailand [41], and red pandas share habitat with giant pandas in Yele Natural Reserve, China [42]. Although Asiatic black bears and red pandas have some common distributional range, this is the first study assessing habitat overlap between the two species. Both reside in temperate forests and prefer similar kinds of food (bamboo shoots) [4,6,16,18–20,22–24,36,59,73,74]. In this study, we found that these two species had highly overlapping habitats. We found a larger proportion of suitable habitat for both species in the buffer zone than in the national park itself, because the buffer zone has more forest area than the core zone [53,64].

Both Asiatic black bear and red panda live in temperate forests [6,18–20,22–24]. As in previous studies, we found that temperate forests covered a large portion of habitat of both Asiatic black bear and red panda. Both conifer and broadleaved species are common in the study area [54,64], but conifer forests cover the largest portion of the study area, as well as the largest portion of the habitat of both Asiatic black bear and red panda (**Table 2**). Additionally, both species face anthropogenic pressure throughout their range [4,16,29]. We found anthropogenic activities in the suitable habitat of both species in our study area (**Table 2**), including 24 km^2^ agricultural land and one km^2^ of built-up area inside the overlapped suitable habitat.

One caveat to our findings is that MaxEnt, the method we used to identify the habitats of Asiatic black bears and red pandas in this study, can only model the potential habitat of the species but not the realized habitat [58]. It also has lower performance for highly detectable species [75]. However, as MaxEnt needs only a few presence points, [43] it is useful for rare species that do not have a large number of presence points available. This software also deletes duplicate presence points (i.e. more than one presence point in a single grid) to decrease the effect of spatial autocorrelation.

### Conclusion

Both Asiatic black bears and red pandas used similar habitat types (i.e., conifer forest) in the study area. Their habitats were highly overlapped, indicating that they can co-exist in the same area. Because the habitats of both species face similar anthropogenic pressures, and most of the habitat of both species was inside the buffer zone of the national park, the government of Nepal and conservation partners can protect both species by conducting conservation efforts in the buffer zone.

Although this study covers only one national park and its buffer zone, it is a first step in describing habitat overlap between the Asiatic black bear and red panda and identifying their important habitat in Himalaya. Larger level studies at the national/regional scale are needed in order to generalize these results.

## Supporting information

S1 Table Species occurrence points. (XLSX)

## References

[1] GoN. Nepal fifth national report to the convention on biological diversity. Vol. March, Governmet of Nepal, Ministry of Forests and Soil Conservation. 2014.

[2] Aryal A, Panthi S, Barraclough RK, Bencini R, Adhikari B, Ji W, et al Habitat selection and feeding ecology of dhole (*Cuon alpinus*) in the Himalayas. J Mammal. 2015;96(1):47–53. 10.1093/jmammal/gyu001

[3] Kandel P, Gurung J, Chhetri N, Ning W, Sharma E. Biodiversity research trends and gap analysis from a transboundary landscape, Eastern Himalayas. J Asia-Pacific Biodivers. 2016;9(1):1–10. 10.1016/j.japb.2015.11.002

[4] Garshelis D, Steinmetz R. Ursus thibetanus. (errata version published in 2017). The IUCN Red List of Threatened Species. 2016. http://www.iucnredlist.org/details/22824/0

[5] CITES. Appendices I, II and III. Convention on international trade in endangered species of wild fauna and flora. 2017. 10.1159/000459796 712806

[6] Chhetri M. Distribution and abundance of Himalayan black bear and brown bear conflict in Manaslu conservation area, Nepal. National Trust for Nature Conservation‐Manaslu Conservation Area Project, Nepal. 2013.

[7] Bista R, Aryal A. Status of the Asiatic black bear *Ursus thibetanus* in the south eastern region of the Annapurna Conservation Area, Nepal. Zool Ecol. 2013;23(1):83–87. 10.1080/21658005.2013.774813

[8] Ali A, Zhou Z, Waseem M, Khan MF, Ali I, Asad M, et al An assessment of food habits and altitudinal distribution of the Asiatic black bear (*Ursus thibetanus*) in the Western Himalayas, Pakistan. J Nat Hist. 2017;51(11–12):689–701. 10.1080/00222933.2017.1303097

[9] Escobar LE, Awan MN, Qiao H. Anthropogenic disturbance and habitat loss for the red-listed Asiatic black bear (*Ursus thibetanus*): Using ecological niche modeling and nighttime light satellite imagery. Biol Conserv. 2015;191:400–407. 10.1016/j.biocon.2015.06.040

[10] Ahmadzadeh F, Liaghati H, Hassanzadeh Kiabi B, Mehrabian AR, Abdoli A, Mostafavi H. The status and conservation of the Asiatic black bear in Nikshahr County, Baluchistan District of Iran. J Nat Hist. 2008;42(35–36):2379–2387. 10.1080/00222930802262741

[11] Jamtsho Y, Wangchuk S. Assessing patterns of human Asiatic black bear interaction in and around Wangchuck Centennial National Park, Bhutan. Glob Ecol Conserv. 2016; 8:183–189. 10.1016/j.gecco.2016.09.004

[12] Charoo SA, Sharma LK, Sathyakumar S. Asiatic black bear–human interactions around Dachigam National Park, Kasmir, India. Ursus. 2011;22(2):106–113. 10.2192/URSUS-D-10-00021.1

[13] Awan MN, Karamanlidis AA, Awan MS, Nawaz MA, Kabir M. Priliminary survey on Asiatic black bear in kasmir Himalaya, Pakisthan: Implication for preservation. Int J Conserv Sci. 2016;7(3):719–724.

[14] DNPWC. Annual Report (July 2009 to June 2010). Department of National Parks and Wildlife Conservation, Nepal. 2010.

[15] Acharya KP, Paudel PK, Neupane PR, Kohl M. Human-wildlife conflicts in Nepal: Patterns of human fatalities and injuries caused by large mammals. PLoS One. 2016;11:e0161717 10.1371/journal.pone.016171715 27612174 PMC5017643

[16] Glatston A, Wei F, Zaw T and, Sherpa A. Ailurus fulgens. 2015. The IUCN Red List of Threatened Species. http://www.iucnredlist.org/details/714/0

[17] GoN. National parks and wildlife conservation act. Nepal: Government of Nepal, Nepal law commission. 1973.

[18] Bhatta M, Shah KB, Devkota B, Paudel R, Panthi S. Distribution and habitat preference of red panda (*Ailurus fulgens fulgens*) in Jumla district, Nepal. Open J Ecol. 2014;4(15):989–1001. 10.4236/oje.2014.415082

[19] Chakraborty R, Nahmo LT, Dutta PK, Srivastava T, Mazumdar K, Dorji D. Status, abundance, and habitat associations of the red panda (*Ailurus fulgens*) in Pangchen Valley, Arunachal Pradesh, India. Mammalia. 2015;79(1):25–32. 10.1515/mammalia-2013-0105

[20] Dorji S, Vernes K, Rajaratnam R. Habitat correlates of the red panda in the temperate forests of Bhutan. PLoS One. 2011;6(10):e26483 10.1371/journal.pone.0026483 22039497 PMC3198399

[21] Panthi S. Feeding ecology, habitat preference and distribution of red panda (Ailurus fulgens fulgens) in Dhopatan hunting reserve, Nepal. BSc thesis. Tribhuvan university, instrtute of forestry, Pokhara, Nepal; 2011.

[22] Panthi S, Aryal A, Raubenheimer D, Lord J, Adhikari B. Summer diet and distribution of the red panda (*Ailurus fulgens fulgens*) in Dhorpatan hunting reserve, Nepal. Zool Stud. 2012;51(5):701–709.

[23] Pradhan S, Saha GK, Khan JA. Ecology of the red panda *Ailurus fulgens* in the Singhalila national park, Darjeeling, India. Biol Conserv. 2001;98(1):11–18. 10.1016/S0006-3207(00)00079-3

[24] Roberts MS, Gittleman JL. Ailurus fulgens. Mamm Species. 1984;222:1–8.

[25] Fei Y, Hou R, Spotila JR, Paladino F V., Qi D, Zhang Z. Metabolic rate of the red panda, *Ailurus fulgens*, a dietary bamboo specialist. PLoS One. 2017;12(3):e0173274 10.1371/journal.pone.0173274 28306740 PMC5356995

[26] Hu Y, Wu Q, Ma S, Ma T, Shan L, Wang X, et al Comparative genomics reveals convergent evolution between the bamboo-eating giant and red pandas. Proc Natl Acad Sci USA. 2017;114(5):201613870 10.1073/pnas.1613870114 28096377 PMC5293045

[27] Sharma HP, Swenson JE, Belant JL. Seasonal food habits of the red panda (*Ailurus fulgens*) in Rara national park, Nepal. Hystrix. 2014;25(1):47–50. 10.4404/hystrix-25.1-9033

[28] Lewis M. Birth and mother rearing of Nepalese red pandas *Ailurus fulgens fulgens* at the Taronga Conservation Society Australia. Int Zoo Yearb. 2011;45(1):250–258. 10.1111/j.1748-1090.2011.00135.x

[29] Panthi S, Khanal G, Acharya KP, Aryal A, Srivathsa A. Large anthropogenic impacts on a charismatic small carnivore: Insights from distribution surveys of red panda *Ailurus fulgens* in Nepal. PLoS One. 2017;12(7):e0180978 10.1371/journal.pone.0180978 28708881 PMC5510994

[30] Dendup P, Cheng E, Lham C, Tenzin U. Response of the endangered red panda *Ailurus fulgens fulgens* to anthropogenic disturbances, and its distribution in Phrumsengla national park, Bhutan. Oryx. 2017;51(4):701–708. 10.1017/S0030605316000399

[31] Sharma HP, Belant JL, Swenson JE. Effects of livestock on occurrence of the Vulnerable red panda *Ailurus fulgens* in Rara national park, Nepal. Oryx. 2014;48(2):228–231. 10.1017/S0030605313001403

[32] Dorji S, Rajaratnam R, Vernes K. The vulnerable red panda *Ailurus fulgens* in Bhutan: distribution, conservation status and management recommendations. Oryx. 2012;46:536–543. 10.1017/S0030605311000780

[33] Wei F, Feng Z, Wang Z, Hu J. Current distribution, status and conservation of wild red pandas *Ailurus fulgens* in China. Biol Conserv. 1999;89(3):285–291. 10.1016/S0006-3207(98)00156-6

[34] Mallick JK. Status of red panda *Ailurus fulgens* in Neora Valley National Park. Small Carniv Conserv. 2010;43:30–36.

[35] Yonzon PB, Hunter ML. Cheese, tourists, and red pandas in the Nepal Himalayas. Conserv Biol. 1991;5(2):196–202. 10.1111/j.1523-1739.1991.tb00124.x

[36] Huygens OC, Miyashita T, Dahle B, Carr M, Izumiyama S, Sugawara T, Hayashi H. Diet and feeding habits of Asiatic black bears in the Northern Japanese Alps. Ursus. 2003;14: 236–245.

[37] Wu W, Li Y, Hu Y. Simulation of potential habitat overlap between red deer (*Cervus elaphus*) and roe deer (*Capreolus capreolus*) in northeastern China. PeerJ. 2016;4: e1756 10.7717/peerj.1756 27019775 PMC4806631

[38] Fleishman ER, Murphy DD, Brussard PP. A new method for selection of umbrella species for conservation planning. Ecol. Appl. 2000; 10 (2): 569–579. 10.1890/1051-0761(2000)010[0569:ANMFSO]2.0.CO;2

[39] Noss RF, Quigley HB, Hornocker MG, Merrill T, Paquet PC. Conservation biology and carnivore conservation in the Rocky Mountains. Conserv. Biol. 1996; 10 (4): 949–963. 10.1046/j.1523-1739.1996.10040949.x

[40] Bista D, Shrestha S, Sherpa P, Thapa GJ, Kokh M, Lama ST, et al Distribution and habitat use of red panda in the Chitwan-Annapurna Landscape of Nepal. PLoS One. 2017;12(10):e0178797 10.1371/journal.pone.0178797 29020020 PMC5636060

[41] Steinmetz R, Garshelis DL, Chutipong W, Seuaturien N. The shared preference niche of sympatric Asiatic black bears and sun bears in a tropical forest mosaic. PLoS One. 2011;6(1): e14509 10.1371/journal.pone.0014509 21283792 PMC3024313

[42] Wei F, Feng Z, Wang Z, Hu J. Habitat use and separation between the giant panda and the red panda. J Mammal. 2000;81(2):448–455. 10.1644/1545-1542(2000)081<0448:HUASBT>2.0.CO;2

[43] Phillips SJ, Anderson RP, Schapire RE. Maximum entropy modelling of species geographic distributions. Ecol Modell. 2006;190:231–259. 10.1016/j.ecolmodel.2005.03.026

[44] Elith J, H. Graham C, P. Anderson R, Dudík M, Ferrier S, Guisan A, et al Novel methods improve prediction of species’ distributions from occurrence data. Ecography. 2006;29(2):129–151. 10.1111/j.2006.0906-7590.04596.x

[45] Phillips SJ. A brief tutorial on Maxent. 2017. Available from: http://biodiversityinformatics.amnh.org/open_source/maxent/

[46] Maslo B, Leu K, Faillace C, Weston MA, Pover T, Schlacher TA. Selecting umbrella species for conservation: A test of habitat models and niche overlap for beach-nesting birds. Biol Conserv. 2016;203:233–242. 10.1016/j.biocon.2016.09.012

[47] York P, Evangelista P, Kumar S, Graham J, Flather C, Stohlgren T. A habitat overlap analysis derived from maxent for tamarisk and the south-western willow flycatcher. Front Earth Sci. 2011;5(2):120–129. 10.1007/s11707-011-0154-5

[48] Johnson SA, Ober HK, Adams DC. Are keystone species effective umbrellas for habitat conservation? A spatially explicit approach. J Nat Conserv. 2017;37:47–55. 10.1016/j.jnc.2017.03.003

[49] Trotta-Moreu N, Lobo JM. Deriving the species richness distribution of Geotrupinae (Coleoptera: Scarabaeoidea) in Mexico from the overlap of individual model predictions. Environ Entomol. 2010;39(1):42–49. 10.1603/EN08179 20146838

[50] Boubli JP, De Lima MG. Modeling the geographical distribution and fundamental niches of *Cacajao* spp. and *Chiropotes israelita* in Northwestern Amazonia via a maximum entropy algorithm. Int J Primatol. 2009;30:217–228. 10.1007/s10764-009-9335-4

[51] Brambilla M, Bassi E, Bergero V, Casale F, Chemollo M, Falco R, et al Modelling distribution and potential overlap between Boreal Owl *Aegolius funereus* and Black Woodpecker *Dryocopus martius*: implications for management and monitoring plans. Bird Conserv Int. 2013;23(4):502–511. 10.1017/S0959270913000117

[52] DNPWC. Annual Report (July 2011-July 2012). Department of National Parks and Wildlife Conservation, Nepal. 2012.

[53] DNPWC. Makalu-Barun National Park. Department of National Parks and Wildlife Conservation. 2017.

[54] Shrestha TB. Development of Ecology of the Arun River Basin in Nepal. The International Centre for Integrated Mountain Development, Nepal 1989.

[55] ESRI. ArcGIS Desktop: Release 10.5. Environmental systems research Redlands, California, USA 2017.

[56] Manzoor SA, Griffiths G, Lukac M. Species distribution model transferability and model grain size–finer may not always be better. Sci. Rep. 2018; 8, 7168 10.1038/s41598-018-25437-1 29740002 PMC5940916

[57] Wang R, Li Q, He S, Liu Y, Wang M, Jiang G. Modeling and mapping the current and future distribution of *Pseudomonas syringae pv*. *actinidiae* under climate change in China. PLoS One. 2018; 13 (2): e0192153 10.1371/journal.pone.0192153 29389964 PMC5794145

[58] Panthi S. Predicting current and future habitat suitability for red pandas in Nepal. MSc thesis. University of Twente,faculty of geoinformation and earth observation, Enschede, Netherlands; 2018.

[59] Dasgupta S, Choudhury P, Ashraf NVK, Bhattacharjee PC, Kyarong S. Food preference of rehabilitated Asiatic black bear cubs in lowland tropical forests of northeast India. Asian J Conserv Biol. 2015;4(1):20–25.

[60] Wang T, Skidmore AK, Toxopeus AG, Liu X. Understory bamboo discrimination using a winter image. Photogramm Eng Remote Sens. 2009;75(1):37–47. doi: 10.14358/PERS.75.1.37

[61] Sun Y. Reassessing giant panda habitat with satellite-derived bamboo information: A case study in the Qinling Mountains, China. MSc thesis. University of Twente, faculity of geo-information science and earth observation, Enschede, Netherlands; 2011.

[62] Hansen MC, Potapov PV, Moore R, Hancher M, Turubanova SA, Tyukavina A. High-Resolution Global Maps of 21st-Century Forest Cover Change. Science. 2013;342:850–853. 10.1126/science.1244693 24233722

[63] Byers AC. Historical and contemporary human disturbance in the upper Barun valley, Makalu-Barun national park and conservation area, east Nepal. Mt. Res. Dev. 1996; 16: 235–247. https://doi.org/DOI:10.2307/3673946

[64] DNPWC. Protected areas of Nepal. Department of national parks and wildlife conservation, Kathmandu, Nepal; 2017.

[65] Uddin K, Shrestha HL, Murthy MSR, Bajracharya B, Shrestha B, Gilani H, et al Development of 2010 national land cover database for the Nepal. J Environ Manage. 2015;148:82–90. 10.1016/j.jenvman.2014.07.047 25181944

[66] Barbet-Massin M, Jiguet F, Albert CH, Thuiller W. Selecting pseudo-absences for species distribution models: how, where and how many? Methods Methods Ecol. Evol. 2012; 3(2):327–338. 10.1111/j.2041-210X.2011.00172.x

[67] Wiley EO, McNyset KM, Peterson AT, Robins CR, Stewart AM. Niche modeling and geographic range predictions in the marine environment using a machine-learning algorithm. Oceanography. 2003;16(3):120–127. 10.5670/oceanog.2003.42

[68] Pearce J, Ferrier S. Evaluating the predictive performance of habitat models developed using logistic regression. Ecol Modell. 2000;133(3):225–245. 10.1016/S0304-3800(00)00322-7

[69] Lobo JM, Jiménez-valverde A, Real R. AUC: a misleading measure of the performance of predictive distribution models. Glob Ecol Biogeogr. 2008;17:145–151. 10.1111/j.1466-8238.2007.00358.x

[70] Merow C, Smith MJ, Silander JA. A practical guide to MaxEnt for modeling species’ distributions: What it does, and why inputs and settings matter. Ecography. 2013;36(10):1058–1069. 10.1111/j.1600-0587.2013.07872.x

[71] Allouche O, Tsoar A, Kadmon R. Assessing the accuracy of species distribution models: prevalence, kappa and the true skill statistic (TSS). J Appl Ecol. 2006;43:1223–1232. 10.1111/j.1365-2664.2006.01214.x

[72] Liu C, White M, Newell G. Selecting thresholds for the prediction of species occurrence with presence-only data. J Biogeogr. 2013;40:778–789. 10.1111/jbi.12058

[73] Panthi S, Coogan SCP, Aryal A, Raubenheimer D. Diet and nutrient balance of red panda in Nepal. Sci Nat. 2015;102:54 10.1007/s00114-015-1307-2 26315537

[74] Thapa A, Hu Y, Wei F. The endangered red panda (*Ailurus fulgens*): Ecology and conservation approaches across the entire range. Biol Conserv. 2018;220:112–121. 10.1016/j.biocon.2018.02.014

[75] Rota CT, Fletcher RJ, Evans JM, Hutto RL. Does accounting for imperfect detection improve species distribution models? Ecography. 2011; 34: 659–670. https://doi:10.1111/j.1600-0587.2010.06433.x

